# The Capping Domain in RalF Regulates Effector Functions

**DOI:** 10.1371/journal.ppat.1003012

**Published:** 2012-11-15

**Authors:** Eric Alix, Laurent Chesnel, Brad J. Bowzard, Aimee M. Tucker, Anna Delprato, Jacqueline Cherfils, David O. Wood, Richard A. Kahn, Craig R. Roy

**Affiliations:** 1 Department of Microbial Pathogenesis, Yale University School of Medicine, Boyer Center for Molecular Medicine, New Haven, Connecticut, United States of America; 2 Department of Biochemistry, Emory University School of Medicine, Atlanta, Georgia, United States of America; 3 Department of Microbiology and Immunology, University of South Alabama, Mobile, Alabama, United States of America; 4 Laboratoire d'Enzymologie et Biochimie Structurales, Centre de Recherche de Gif, CNRS Bat 34, CNRS, Gif sur Yvette, France; Purdue University, United States of America

## Abstract

The *Legionella pneumophila* effector protein RalF functions as a guanine nucleotide exchange factor (GEF) that activates the host small GTPase protein ADP-ribosylation factor (Arf), and recruits this host protein to the vacuoles in which this pathogen resides. GEF activity is conferred by the Sec7 domain located in the N-terminal region of RalF. Structural studies indicate that the C-terminal region of RalF makes contacts with residues in the Sec7 domain important for Arf interactions. Theoretically, the C-terminal region of RalF could prevent nucleotide exchange activity by blocking the ability of Arf to interact with the Sec7 domain. For this reason, the C-terminal region of RalF has been termed a capping domain. Here, the role of the RalF capping domain was investigated by comparing biochemical and effector activities mediated by this domain in both the *Legionella* RalF protein (LpRalF) and in a RalF ortholog isolated from the unrelated intracellular pathogen *Rickettsia prowazekii* (RpRalF). These data indicate that both RalF proteins contain a functional Sec7 domain and that the capping domain regulates RalF GEF activity. The capping domain has intrinsic determinants that mediate localization of the RalF protein inside of host cells and confer distinct effector activities. Localization mediated by the capping domain of LpRalF enables the GEF to modulate membrane transport in the secretory pathway, whereas, the capping domain of RpRalF enables this bacterial GEF to modulate actin dynamics occurring near the plasma membrane. Thus, these data reveal that divergence in the function of the C-terminal capping domain alters the *in vivo* functions of the RalF proteins.

## Author Summary

*Legionella pneumophila* and *Rickettsia prowazekii* are two pathogenic intracellular bacteria, phylogenetically distant and presenting different intracellular lifestyles. Interestingly, both organisms encode a protein called RalF, which in *Legionella* has been shown to be an effector protein that functions as a guanine nucleotide exchange factor (GEF) for the Arf family of eukaryotic GTPases. We show that the Sec7 domains in both proteins have a similar enzymatic ability to activate Arf1, however, the two proteins differ in their effector functions inside mammalian cells. The *Legionella* RalF protein targets secretory transport functions, whereas, the *Rickettsia* RalF protein modulates actin dynamics at the plasma membrane. These differences in RalF effector activities are mediated by a C-terminal domain that directs localization and interaction with host cell determinants. These data reveal important properties of the RalF protein that provide insight into differential roles for Arf during infection by *Legionella* and *Rickettsia*, and also provide a model for investigating how intracellular localization of structurally similar Arf GEFs enables a protein to regulate functionally distinct host cell processes.

## Introduction

The Arf family of small GTPases plays an important role in regulating transport of membranes and proteins inside of eukaryotic cells [1], [2]. Mammalian cells encode six different Arf proteins that belong to three distinct classes [3]. Arf1, Arf2 and Arf3 comprise the class I family, Arf4 and Arf5 comprise the class II family, and Arf6 is the sole member of the class III family [4]. Arfs cycle between an active GTP-bound state and an inactive GDP-bound conformation. Guanine nucleotide exchange factors (GEFs) function in Arf activation by stimulating the exchange of GDP for GTP [5], and inactivation of Arf functions depends on GTP hydrolysis stimulated by GTPase activating proteins (GAPs) [6]. Proteins that activate Arf contain a highly conserved Sec7 domain that is sufficient for GEF activity *in vitro* [5]. The human genome encodes at least 15 different proteins containing a Sec7 domain. The diversity in the GEFs in relation to the limited number of Arfs is important because GEFs confer spatial regulation of Arf activation and also serve as platforms that assist in recruiting other cellular factors involved in Arf-dependent biological processes [7]–[10].

Intracellular bacteria have evolved strategies to subvert mammalian cell functions during infection. Several species possess sophisticated secretion systems that allow them to inject proteins that specifically modulate the function of eukaryotic proteins [11], [12]. Because of their highly conserved structures and their ability to regulate numerous cellular processes, small GTPases are a common target for pathogen manipulation [13], [14]. *Legionella pneumophila* is a facultative intracellular bacterium that subverts the host secretory pathway to build a vacuole presenting endoplasmic reticulum (ER) determinants, where it can replicate [15]–[19]. *Legionella* has a type IV secretion system called Dot/Icm that translocates bacterial effectors required for the formation of the *Legionella*-containing vacuole (LCV) [16]. The RalF protein is a translocated effector that contains an N-terminal Sec7 domain [20], [21]. RalF is essential for the recruitment of Arf1 to the LCV during infection [21].

Arfs are recruited to the LCV during infection by a mechanism that requires RalF amino acid E103, a conserved residue required for the GEF activity displayed by Sec7 domain-containing proteins [22]. Additionally, RalF overexpression in yeast results in a severe growth defect by a mechanism that is dependent on this conserved E103 residue in the Sec7 domain [23]. These data indicate that GEF activity *in vivo* is important for RalF function. Structural studies confirmed that RalF has an N-terminal Sec7 domain that is structurally similar to eukaryotic Sec7 domains [22]. The structure also revealed that the RalF C-terminal region contains a distinct globular domain that makes extensive electrostatic and hydrophobic contacts with the Sec7 domain which block the Arf docking and catalytic sites [22]. These two domains are connected by a short surface exposed linker which imparts flexibility to the molecule. Because this C-terminal domain would prevent Arf from binding to the Sec7 domain, it is believed that the solved structure represents an auto-repressed conformation of RalF. This would imply that *in vivo*, the C-terminal region, which has been called the capping domain, would reorient and disengage from the Sec7 domain to facilitate functional interactions between Arf and the GEF domain.

After translocation by *Legionella* into the host cytosol, RalF is found associated with the cytosolic surface of the LCV membrane [21]. RalF structural data does not reveal any membrane interaction determinants that would be predicted to mediate an association with the LCV [22]. The capping domain of RalF, however, has structural similarities with a domain found in eukaryotic proteins that assist in creating membrane vesicles through the recruitment of coat proteins [22]. These data suggest that the capping domain might play a role in regulating RalF function by a process that could involve interactions with determinants on the LCV membrane.

**Figure 1:**
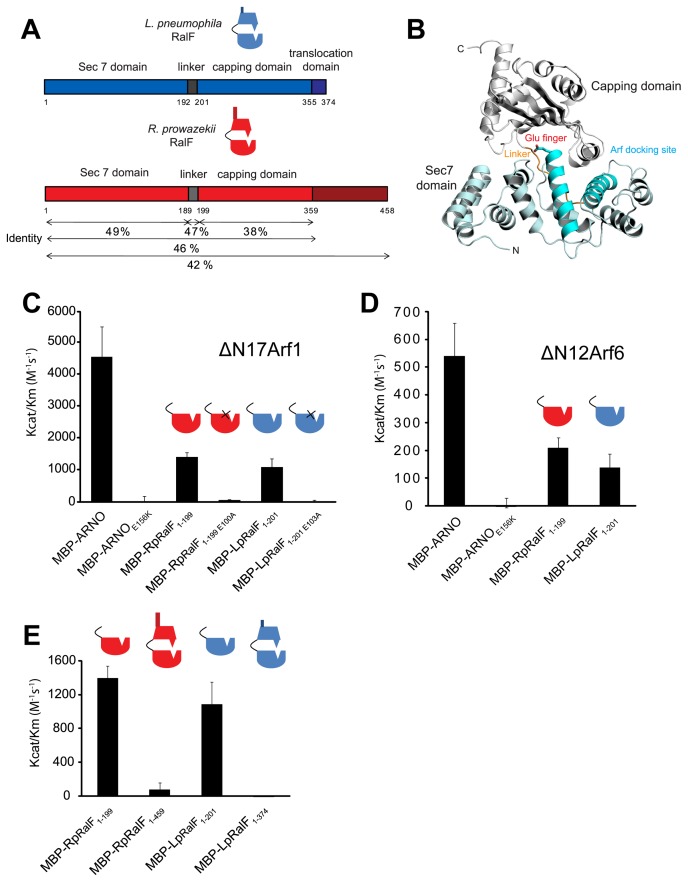
*Legionella* and *Rickettsia* RalF proteins are autoinhibited by their capping domain *in vitro*. **A**) Alignment of the *Legionella* and *Rickettsia* RalF full length proteins. **B**) Structural organization of LpRalF protein. Ribbon rendering showing LpRalF Sec7 domain (blue), linker (orange) and capping domain (white) (Protein Data Bank accession code 1XSZ, image generated with PyMOL (http://pymol.sourceforge.net)) [22]. The Sec7 catalytic glutamic acid side chain is represented as a stick with oxygen atoms shown in red. **C**) LpRalF and RpRalF Sec7 domains activate His-ΔN17Arf1 *in vitro*. Efficiency of His-ΔN17Arf1 nucleotide exchange catalyzed by the indicated MBP-tagged proteins. K_cat_/K_m_ values were obtained as described in Materials and Methods. Average and standard deviation are calculated from three independent experiments. **D**) Efficiency of His-ΔN12Arf6 nucleotide exchange catalyzed by MBP-tagged LpRalF and RpRalF Sec7 domains. K_cat_/K_m_ values were obtained as described in Materials and Methods. Average and standard deviation are calculated from three independent experiments. **E**) The RalF capping domain regulates GEF activity. Comparison of K_cat_/K_m_ values for His-ΔN17Arf1 nucleotide exchanged catalyzed by the Sec7 domain and the full length RalF proteins. Average and standard deviation are calculated from three independent experiments.

A RalF ortholog is encoded by the unrelated intracellular pathogen *Rickettsia prowazekii* [21]. The *L. pneumophila* RalF protein (LpRalF) and the *R. prowazekii* RalF protein (RpRalF) display 46% identity over the regions containing the Sec7 domain and the capping domain (Figure 1A). It has been hypothesized that RpRalF is delivered into the host cytosol by a type IVA secretion system during *R. prowazekii* infection [24]. *R. prowazekii* mediates vacuolar lysis shortly after uptake by mammalian cells and replicates in the host cytosol [24], [25]. Thus, it is unlikely that RpRalF is involved in remodeling of the vacuole in which this pathogen resides, suggesting that it may function differently than LpRalF.

In this study we compare and contrast the *in vitro* and *in vivo* activities displayed by LpRalF and RpRalF in the hope of gaining a better understanding of RalF regulation during infection and the role distinct domains may play in the effector functions of this protein.

## Results

### The LpRalF and RpRalF Sec7 domains have similar catalytic activity

The Sec7 domains at the N-terminus of LpRalF and RpRalF display 49% amino acid identity (Figure 1A), which is higher than the 36% identity between the Sec7 domains from the human exchange factors ARNO and EFA6. This suggested that the RalF Sec7 domains could have similar catalytic activity and Arf specificities. To address these questions, MBP-tagged LpRalF_Sec7_ and RpRalF_Sec7_ were purified and catalytic activities were measured *in vitro* using a mant-GDP release assay. Purified His-ΔN17Arf1 loaded with mant-GDP was incubated with the MBP-tagged GEFs at different concentrations, and the K_cat_/K_m_ of GDP-release mediated by each Sec7 domain was determined (Figure 1C). The host protein ARNO was used as a positive control. The catalytic activity displayed by MBP-LpRalF_Sec7_ and MBP-RpRalF_Sec7_ were similar and roughly 4-fold lower than the catalytic activity determined for MBP-ARNO. Catalytic activity was not detected using MBP-ARNO_E156K_, MBP-RpRalF_Sec7E100A_ or MBP-LpRalF_Sec7E103A_, consistent with the highly conserved glutamic acid residue in the Sec7 active site being essential for GEF activity in all three proteins. To determine the Arf specificity of the RalF Sec7 domains, the rate of GDP-release using purified His-ΔN12Arf6 (Figure 1D) was compared to purified His-ΔN17Arf1 (Figure 1C). As reported previously [26], MBP-ARNO displayed preferential activity for His-ΔN17Arf1 compared to His-ΔN12Arf6 *in vitro*. Similarly, MBP-LpRalF_Sec7_ and MBP-RpRalF_Sec7_ showed a preference for His-ΔN17Arf1 compared to His-ΔN12Arf6. Thus, the LpRalF and RpRalF Sec7 domains have very similar catalytic activities and preferentially promote the exchange of GDP for GTP on Arf1 *in vitro*.

### The RalF capping domain regulates GEF activity

The RalF crystal structure indicates that interactions at the interface between the Sec7 domain and the C-terminal capping domain would prevent the Sec7 domain from interacting with Arf [22](Figure 1B), suggesting that the capping domain may interfere with the catalytic activity of the Sec7 domain. To test whether the capping domain is involved in regulating GEF activity, the catalytic activity of the full-length RalF proteins from *Legionella* and *Rickettsia* was determined. These data revealed that the catalytic activity of the MBP-LpRalF and MBP-RpRalF proteins was greatly reduced when derivatives containing the capping domain were compared to the derivatives containing only the Sec7 domain, using His-ΔN17Arf1 as substrate (Figure 1E). Thus, as the structural data predict, the capping domain is involved in inhibiting the GEF activity of RalF.

### The RalF capping domain mediates membrane localization in host cells

**Figure 2:**
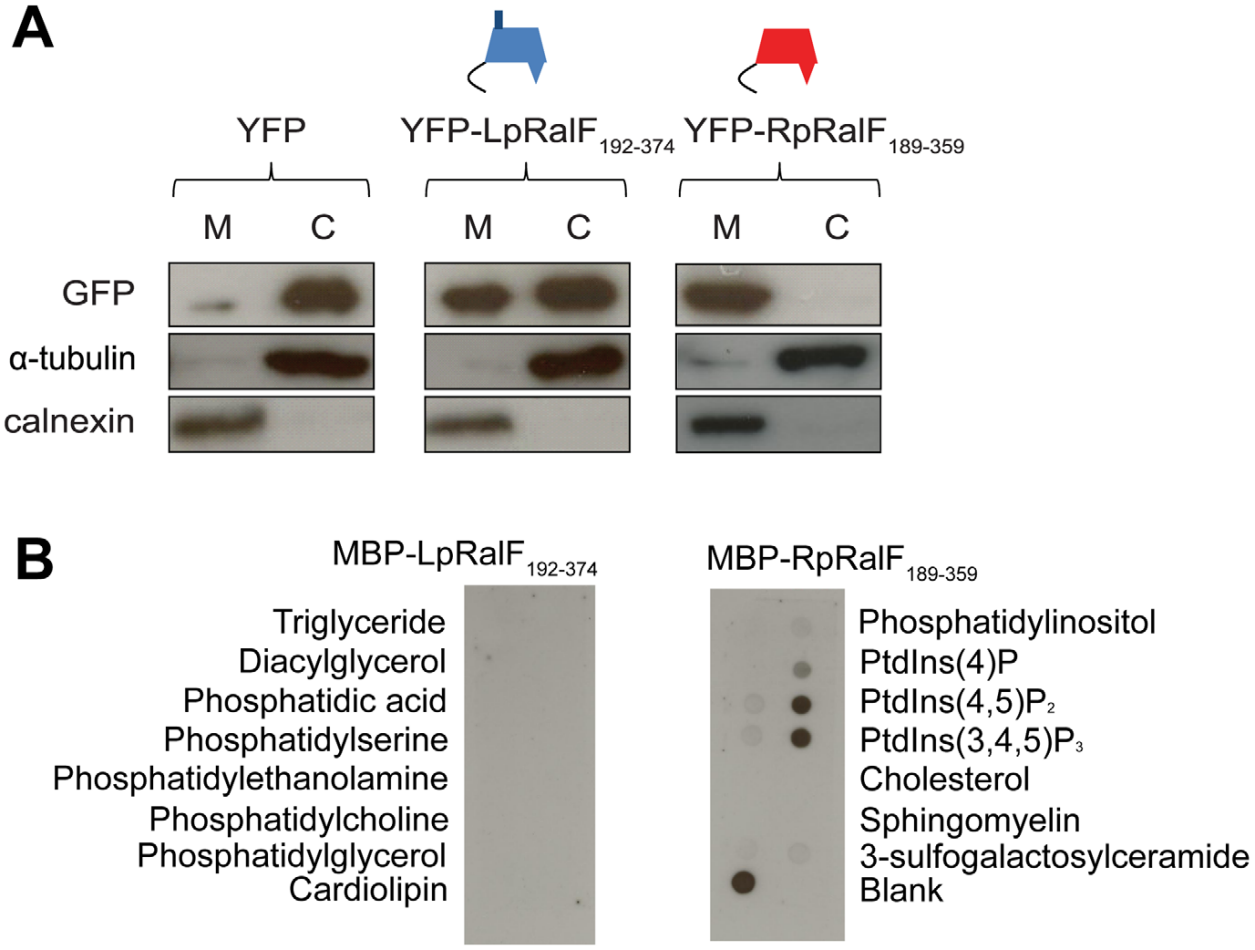
LpRalF and RpRalF capping domains associate with membranes by different mechanisms. **A**) LpRalF and RpRalF capping domains are associated with the cell membrane fraction. HEK293 cells were transfected with YFP-LpRalF_192–374_, YFP-RpRalF_189–359_ or YFP alone. 24 hours after transfection, cells were lysed and centrifuged at 100,000 g for 1 h. The pellet (M) fraction was resuspended in a volume identical to the supernatant (C) fraction. Samples were blotted with GFP, calnexin (membrane marker) or α-tubulin (cytosol marker) antibodies. **B**) Protein-lipid overlay assay. The binding of MBP-LpRalF and RpRalF capping domains to indicated lipids immobilized on nitrocellulose membranes was analyzed using an anti-MBP antibody.

To better understand how RalF may be regulated in host cells by the capping domain, the capping domains were fused to YFP and the fluorescent proteins were produced in Human Embryonic Kidney (HEK293) cells. Protein localization was first assessed by cell fractionation. YFP-LpRalF_192–374_ and YFP-RpRalF_189–359_ were both detected in membrane fractions (Figure 2A), suggesting that the capping domain may be important for membrane localization of the RalF protein. To further define the mechanism by which RalF is able to interact with host organelles, we evaluated the binding of MBP-tagged LpRalF and RpRalF capping domains with different lipids arrayed on a solid matrix (Figure 2B). The LpRalF capping domain did not interact preferentially with the lipids on this array, which is consistent with data published previously using the full-length LpRalF [27]. The RpRalF capping domain interacted preferentially with negatively-charged lipids, with a preference for cardiolipin and the phosphoinositides PI(4,5)P_2_ and PI(3,4,5)P_3_. This indicates that LpRalF and RpRalF capping domains have different affinities for host lipids, which could lead to differential subcellular targeting of the effector proteins inside eukaryotic cells.

**Figure 3:**
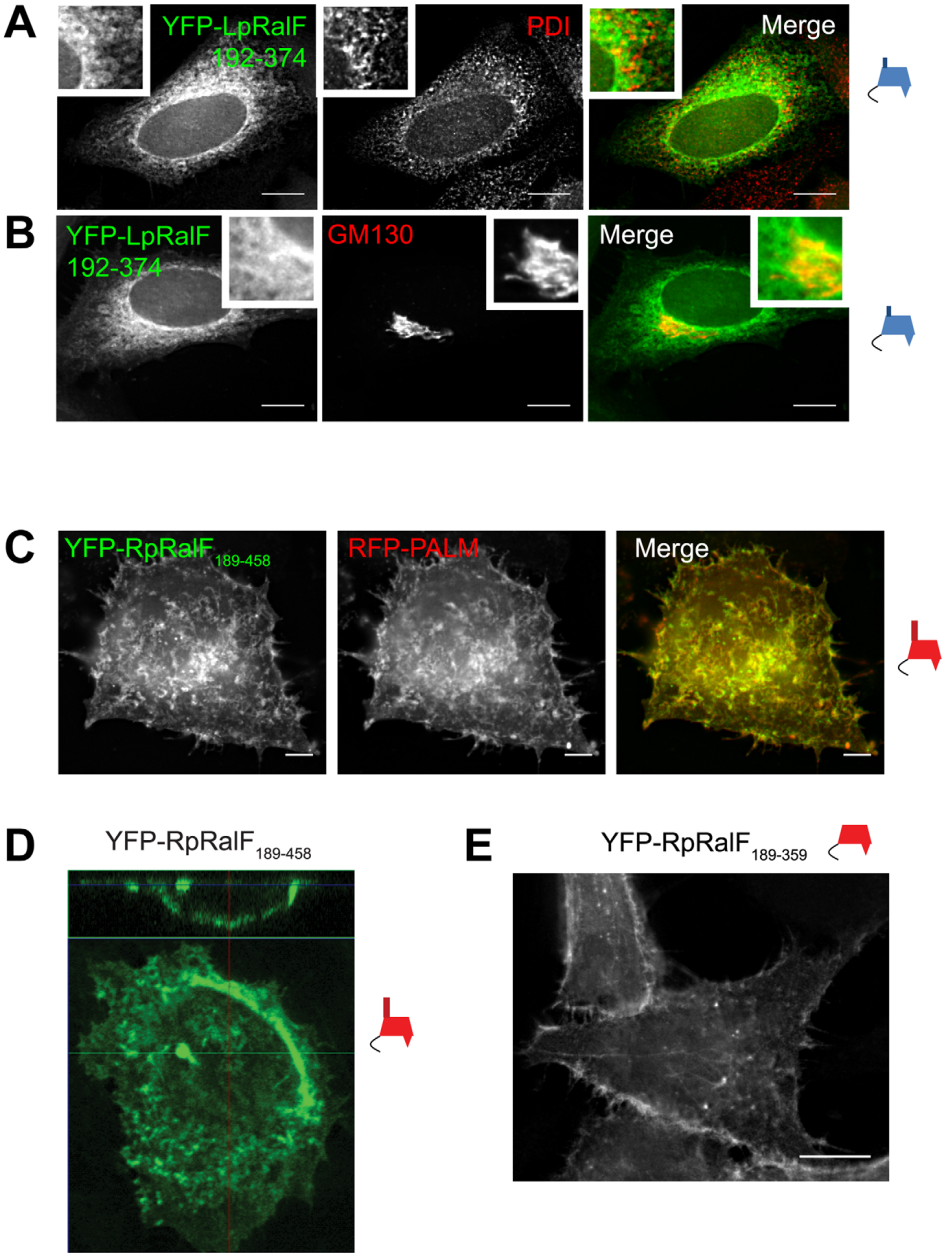
LpRalF and RpRalF capping domains associate with different subcellular compartments. **A**) and **B**) Fluorescence microscopy of HeLa cells transfected with YFP-LpRalF_192–374_. Cells were fixed 24 hours after transfection, then stained with anti-PDI (**A**) or anti-GM130 (**B**) antibodies as indicated. Bar = 10 µm. **C**) Fluorescence microscopy of HeLa cells transfected with YFP-RpRalF_189–458_ and RFP-PALM. Bar = 5 µm. **D**) Confocal microscopy shows that RpRalF_189–458_ localizes at the plasma membrane. **E**) RpRalF capping domain is sufficient to target plasma membrane. Fluorescence microscopy of HeLa cells transfected with YFP-RpRalF_189–359_. Bar = 10 µm.

Fluorescence microscopy was used to localize tagged RalF proteins expressed ectopically in HeLa cells to determine if different membrane-bound organelles were targeted by the RalF capping domains. YFP-LpRalF_192–374_ displayed a reticulate pattern in cells that resembled the ER network (Figure 3A). Overlap was observed between YFP-LpRalF_192–374_ and protein disulfide isomerase (PDI) residing in the host ER. YFP-LpRalF_192–374_ was also enriched in an area near the Golgi apparatus, which was visualized using an antibody specific for the Golgi matrix protein GM130 (Figure 3B). These data suggest that the capping domain of LpRalF has determinants that enable this protein to associate with membranes of the host early secretory pathway.

YFP-RpRalF_189–458_ had a dramatically different pattern of localization in mammalian cells when compared to YFP-LpRalF_192–374_ (Figure 3C). The pattern of staining was evenly distributed and more consistent with localization to the plasma membrane, which would correlate with the *in vitro* association of the RpRalF capping domain with the lipids PI(4,5)P_2_ and PI(3,4,5)P_3_, which are enriched at the plasma membrane. Localization of YFP-RpRalF_189–458_ to the plasma membrane was further indicated by colocalization with a palmitoylated RFP protein that labels the plasma membrane (Figure 3C), and by confocal microscopy that demonstrated YFP-RpRalF_189–458_ localization at the periphery of the cell (Figure 3D). Because YFP-RpRalF_189–458_ contains the capping domain and a 100-amino acid C-terminal extension that has no homology to the 20-amino acid C-terminal extension in LpRalF, localization of YFP-RpRalF_189–359_ was examined to determine if the capping domain alone was sufficient for plasma membrane localization. The YFP-RpRalF_189–359_ protein showed a similar pattern of plasma membrane localization (Figure 3E), indicating the capping domain of the RpRalF protein is sufficient for plasma membrane targeting.

### The LpRalF capping domain targets the host secretory pathway

**Figure 4:**
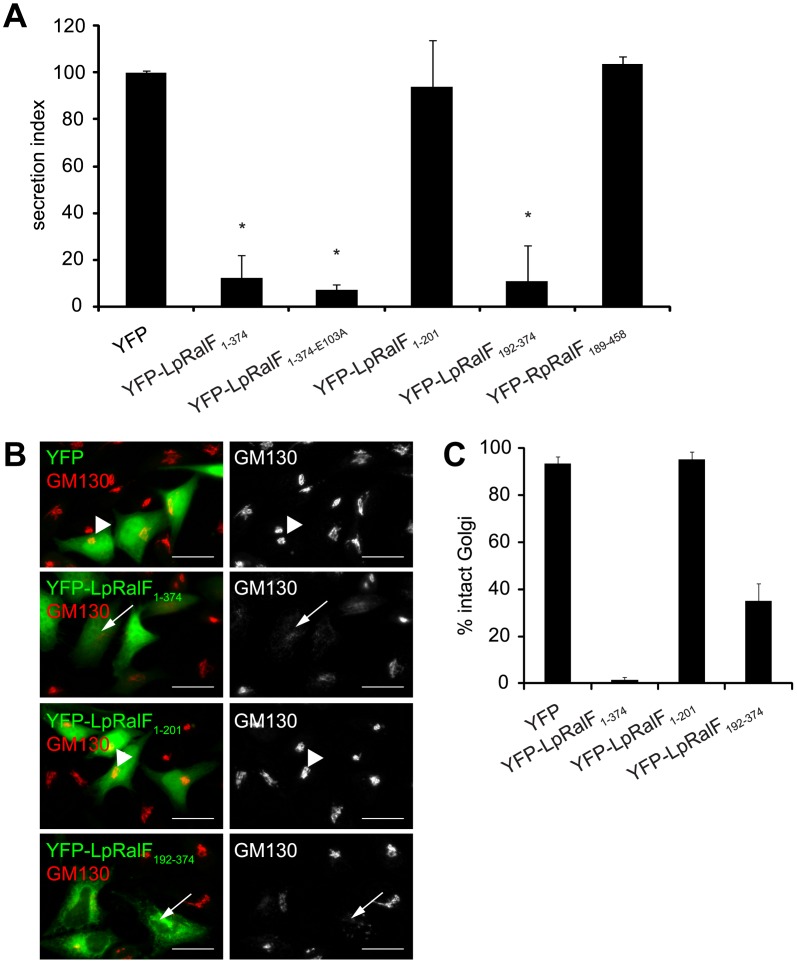
*Legionella* RalF capping domain disrupts secretion and the Golgi apparatus. **A**) LpRalF capping domain disrupts secretion. Vectors encoding the indicated YFP-tagged *L. pneumophila* or *R. prowazekii* RalF constructs were co-transfected into CHO cells along with a plasmid encoding SEAP. Alkaline phosphatase secretion from CHO cells was plotted as the ratio of SEAP in the culture medium to cell-associated SEAP (Secretion Index). Vector alone (YFP) served as a negative control. These data are from at least 3 independent experiments done in triplicate. The results are normalized so the cells expressing the empty plasmid have a secretion value of 100% (* P<0.001). **B**) Fluorescence microscopy of Hela cells ectopically expressing YFP alone, YFP-LpRalF_1–374_, YFP-LpRalF_1–201_ or YFP-LpRalF_192–374_. Cells were fixed 24 hours after transfection, then stained with anti-GM130 antibody (red). Arrows indicate disrupted Golgi, arrowheads indicate intact Golgi. Bar = 25 µm. **C**) Golgi disruption was quantified in cells expressing YFP alone, YFP-LpRalF_1–374_, YFP-LpRalF_1–201_ or YFP-LpRalF_192–374_. These data were obtained from three independent experiments. Standard deviations are represented.

Previous studies showed that expression of the LpRalF protein in mammalian cells was sufficient to interfere with protein secretion [22], and it was possible that dysregulation of Arf activation by the Sec7 domain of RalF could account for this phenotype. Derivatives of LpRalF and RpRalF were produced in mammalian cells to better understand the mechanism by which RalF interferes with the host secretory pathway. These data revealed that production of full-length YFP-LpRalF_1–374_ in mammalian cells blocked secretion of an alkaline phosphatase enzyme into the culture supernatant and resulted in fragmentation of the Golgi apparatus (Figure 4A–4C), consistent with previous data. The Sec7 domain alone in YFP-LpRalF_1–201_ was not sufficient to disrupt the host secretory pathway, and the YFP-LpRalF_E103A_ mutant, defective in GEF activity, retained the ability to disrupt the host secretory pathway. When the Sec7 domain was deleted from LpRalF, the resulting protein, YFP-LpRalF_192–374_, was sufficient to disrupt the host secretory pathway as determined by a defect in both the secretion of an alkaline phosphatase reporter into the supernatant (Figure 4A) and the assembly of the Golgi apparatus as determined by GM130 staining (Figure 4B and C). In contrast, none of the YFP-RpRalF constructs were able to interfere with the host secretory pathway (Figure 4A and data not shown). Thus, the LpRalF capping domain is necessary and sufficient to interfere with the host early secretory pathway when produced ectopically in mammalian cells, and the finding that RpRalF did not have a similar activity indicates a divergent function associated with the RpRalF capping domain.

### The RpRalF protein modulates actin dynamics

**Figure 5:**
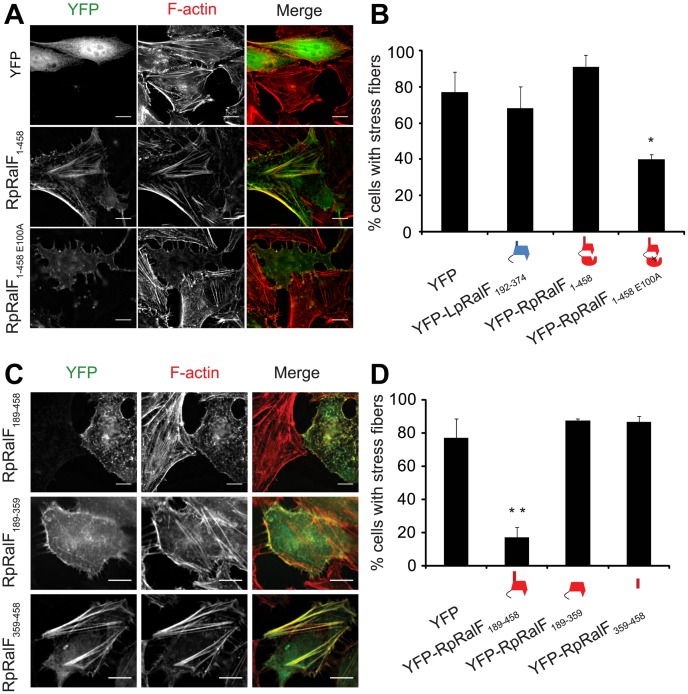
RpRalF modulates actin dynamics. **A**) Hela cells transfected with plasmids encoding YFP, YFP-RpRalF_1–458_ or YFP-RpRalF_1–458E100A_ were fixed 24 hours after transfection, then stained with Texas Red Phalloidin. Bar = 10 µm. **B**) Actin stress fibers disruption quantification. Cells were transfected with indicated YFP-tagged RpRalF or LpRalF constructs. 24 h after transfection, cells were fixed and actin was stained with phalloidin. The proportion of cells containing stress fibers was quantified for each construct. The standard deviation is derived from 3 independent experiments (* P<0.01, compared to YFP alone). **C**) and **D**) The RpRalF capping domain and a proline-rich region direct effector functions. **C**) Localization of ectopically expressed RpRalF_189–458_, RpRalF_189–359_ and RpRalF_359–458_. Hela cells were transfected with YFP-tagged RpRalF_189–458_, RpRalF_189–359_ or RpRalF_359–458_. 24 h after transfection, cells were fixed and stained with Texas Red phalloidin. Bar = 10 µm. **D**) RpRalF_189–359_ and RpRalF_359–458_ domains are both required for stress fibers disruption. Cells were transfected with indicated YFP-tagged RpRalF constructs. 24 h after transfection, cells were fixed and actin was stained with phalloidin. The proportion of cells containing stress fibers was quantified for each construct. The standard deviation is derived from 3 independent experiments (* * P<0.001).

The finding that the RpRalF capping domain localizes to the plasma membrane and does not interfere with secretory transport suggested that this effector targets different host processes compared to LpRalF. Since RpRalF has *in vitro* GEF activity and *in vivo* membrane localization phenotypes that are similar to the host protein ARNO [28], these proteins could have a related function. One of the best-characterized roles for ARNO in mammalian cells involves regulation of the actin cytoskeleton to direct cell migration and endocytosis [5], [29]. To determine if RpRalF modulates actin dynamics, cells producing YFP-RpRalF constructs were analyzed by fluorescence microscopy. These studies revealed that full length YFP-RpRalF colocalizes with actin stress fibers, indicating a possible modulation of the actin cytoskeleton (Figure 5A). Unexpectedly, only 40% of the cells producing the GEF-deficient YFP-RpRalF_E100A_ protein had normal actin stress fibers, as compared to 80% for control cells (Figure 5A and 5B). These data suggest that the GEF-deficient mutant RpRalF_E100A_ acts as a dominant-negative protein that disrupts stress fibers.

### The RpRalF capping domain and a proline-rich region direct effector functions

Given data indicating the LpRalF capping domain is sufficient to disrupt the host secretory pathway, the finding that the catalytically-inactive RpRalF_E100A_ protein could disrupt actin dynamics raised the possibility this dominant-negative activity was mediated by the capping domain. The YFP-RpRalF_189–359_ construct producing only the RpRalF capping domain was analyzed to determine if it interfered with the formation of stress fibers in cells. These data revealed that YFP-RpRalF_189–359_ had no measurable effect on stress fiber formation (Figure 5C and 5D). Because YFP-RpRalF_189–359_ does not contain the RpRalF_359–458_ tail region, it remained possible that stress fiber disruption might require both the capping domain and the tail region. Consistent with this hypothesis, expression of YFP-RpRalF_189–458_ resulted in the disruption of stress fibers. Time-lapse video microscopy showed that production of YFP-RpRalF_189–458_ affected cell adherence and was toxic (Video S1), a phenotype that is induced by other proteins that disrupt actin dynamics. Production of YFP-RpRalF_359–458_ did not have an effect on stress fiber formation (Figure 5C and 5D), indicating the C-terminal tail is necessary for this activity, but not sufficient. Importantly, YFP-RpRalF_359–458_ colocalized with actin-rich stress fibers, indicating this tail region has the ability to interact with host determinants located in these structures. Taken together, these data indicate that RpRalF has two distinct localization domains that work in conjunction to modulate actin dynamics in cells. The capping domain targets RpRalF to the plasma membrane and the tail region interacts with components of the actin cytoskeleton.

### RpRalF inefficiently restores Arf1 recruitment to vacuoles containing *Legionella ΔralF*

**Figure 6:**
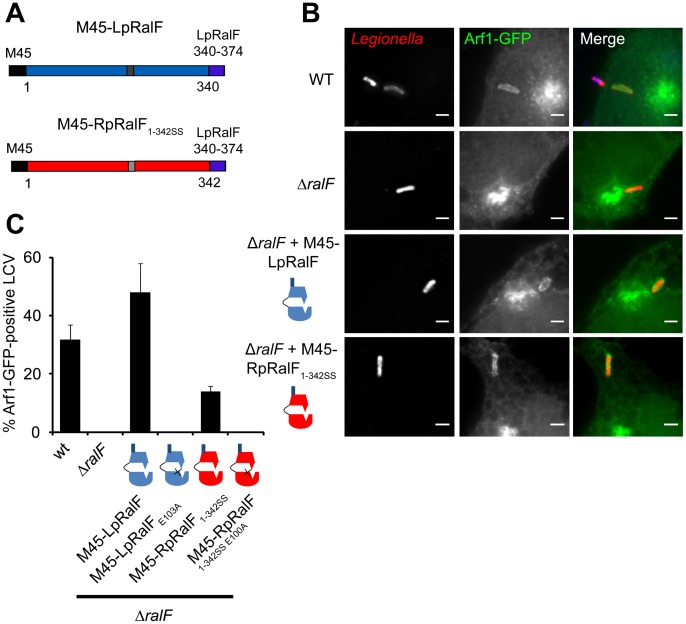
RpRalF inefficiently restores Arf1 recruitment to vacuoles containing *Legionella ΔralF*. **A**) Representation of the constructs used in the complementation experiment. **B**) HEK293 cells stably expressing Arf1-GFP were infected with different strains of *L. pneumophila* (wt, *ΔralF* mutant and *ΔralF* mutant complemented with M45-LpRalF, M45-LpRalF_E103A_, M45-RpRalF_1–342SS_ or M45-RpRalF_1–342SSE100A_,). Cells were fixed 1 h post-infection, extracellular bacteria were stained in blue, and total bacteria in red. Bar = 1 µm. **C**) Quantification of Arf1-GFP recruitment to the LCV by the indicated *L. pneumophila* strains. Represented is the average of 3 experiments where 50 vacuoles were counted. Standard deviations are indicated.

The difference in LpRalF and RpRalF localization mediated by the capping domain suggested that the two proteins would differ in their abilities to mediate recruitment of Arf1 to the vacuole containing *Legionella* when delivered by the Dot/Icm system during infection. To test this hypothesis a translocation-competent derivative of RpRalF with a domain organization similar to the LpRalF protein was generated (Figure 6A). This involved removing the C-terminal “tail” residues (amino acids 343–458) in RpRalF that are adjacent to the capping domain, and replacing these residues with the C-terminal “tail” from LpRalF (amino acids 340–374) that have been shown to encode the signal sequence that mediates translocation of LpRalF by the Dot/Icm system [20]. The resulting gene encoding RpRalF_1–342SS_ was inserted into an expression plasmid in frame with an N-terminal M45 epitope tag and protein expression in a *Legionella* Δ*ralF* strain was measured by immunoblot analysis (Figure S1A). The level of M45-RpRalF_1–342SS_ protein produced in *Legionella* was similar to the isogenic control strain producing M45-LpRalF from the same plasmid.

A HEK293 cell line that stably produces Arf1-GFP was used to evaluate Arf1 recruitment to the LCV. Cells were infected with either wild type *Legionella* (wt) or an isogenic RalF-deficient mutant (Δ*ralF*) producing either M45-LpRalF or M45-RpRalF_1–342SS_. Assays that measured Dot/Icm-mediated delivery of adenylate cyclase-tagged derivatives of these RalF proteins into the host cytosol demonstrated that both LpRalF and RpRalF_1–342SS_ are translocated into the host cytosol to similar levels (Figure S1B). As described previously [21], [22], Arf1-GFP was associated with vacuoles containing wild type *Legionella* 1 h post-infection and was not associated with vacuoles containing the *Legionella* Δ*ralF* strain (Figure 6B and 6C). Production of M45-LpRalF in the *Legionella* Δ*ralF* mutant restored Arf1-GFP recruitment to the vacuole. LpRalF expression from a plasmid enhanced the recruitment of Arf1-GFP to the vacuole compared to the level of recruitment observed when using the control strain producing the chromosomally-encoded LpRalF protein (Figure 6B and 6C). Importantly, recruitment of Arf1-GFP to the vacuole was detected when M45-RpRalF_1–342SS_ was produced in the *Legionella* Δ*ralF* mutant, however, the percentage of vacuoles staining positive for Arf1-GFP was significantly lower than in the control strain producing M45-LpRalF. Substitution mutations that changed the conserved glutamic acid residue in the Sec7 domain to alanine eliminated recruitment of Arf1-GFP to the vacuole for strains producing M45-LpRalF_E103A_ and for strains producing M45-RpRalF_1–342SSE100A_ (Figure 6B and 6C). Thus, GEF activity is required for Arf1 recruitment mediated by M45-RpRalF_1–342SS_. These data suggest that when delivered into host cells by the *Legionella* Dot/Icm system, the Sec7 domain of RpRalF functions in Arf1 activation, however, inefficient recruitment of Arf1 to the LCV is likely the result of differences in activities associated with the capping domain of RpRalF compared to the LpRalF capping domain.

### Chimeric proteins demonstrate that the capping domain confers efficient Arf1 recruitment to the LCV

**Figure 7:**
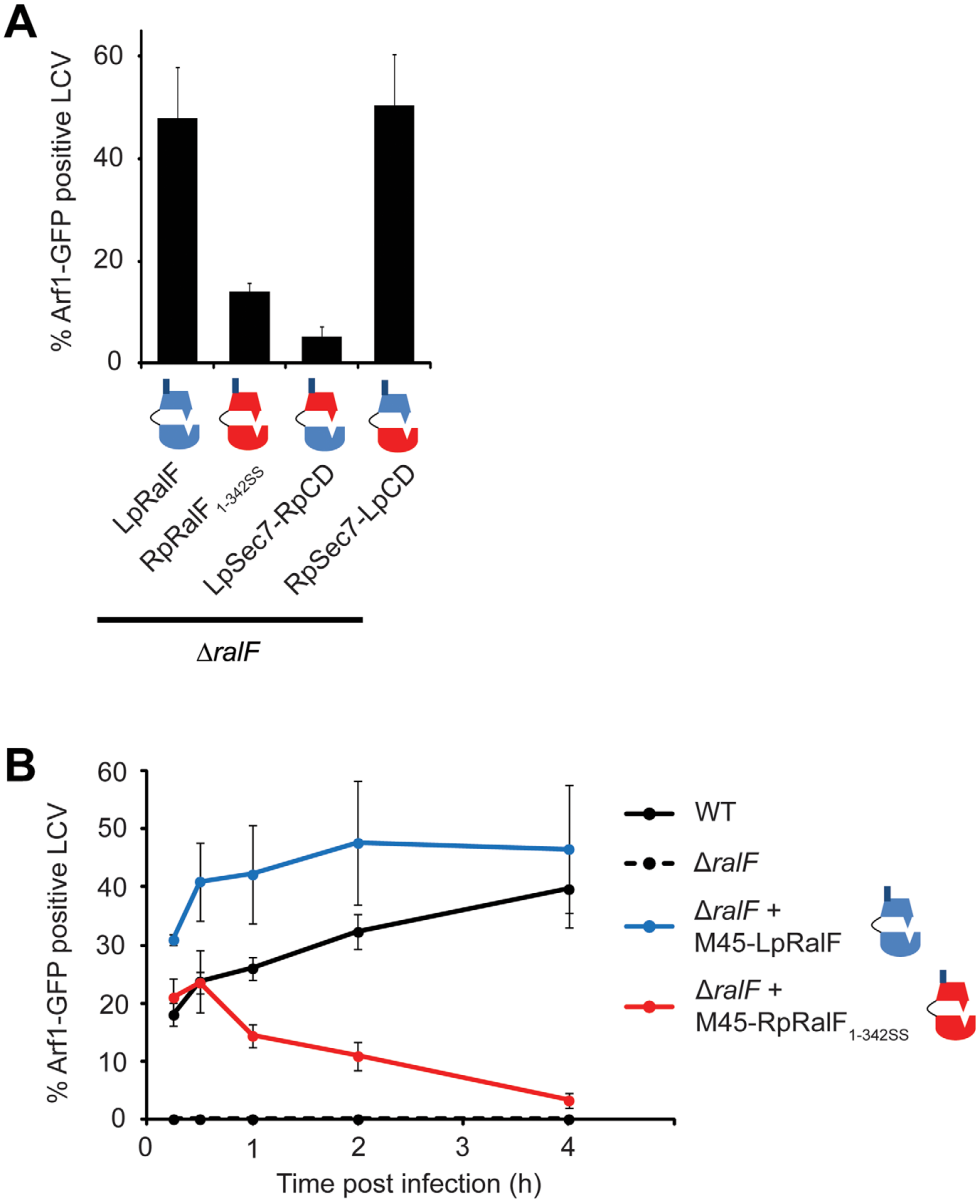
RalF capping domain is the critical determinant for efficient Arf1 recruitment. **A**) HEK293 cells stably expressing Arf1-GFP were infected with *L. pneumophila* Δ*ralF* complemented with the indicated constructs. Arf1-GFP recruitment to the LCV was quantified 1 h post-infection. Represented is the average of 3 experiments where 50 vacuoles were counted. Standard deviations are indicated. **B**) Kinetics of Arf1-GFP recruitment to the LCV by *Legionella* wt, Δ*ralF*, *or* Δ*ralF* expressing M45-LpRalF or M45-RpRalF_1–342SS_. Represented is the average of 3 experiments where 50 vacuoles were counted. Standard deviations are indicated.

To test whether capping domain functions account for the differences in Arf1-GFP recruitment to the LCV observed when using LpRalF or RpRalF proteins, chimeric proteins were constructed having the RalF capping domains fused to a heterologous Sec7 domain. These chimeric proteins were expressed and translocated as efficiently as LpRalF and RpRalF_1–342SS_ proteins (Figure S1). The chimeric M45-RpSec7-LpCD protein having the capping domain from the LpRalF protein fused to the Sec7 domain from RpRalF was as efficient in mediating Arf1-GFP recruitment to the LCV as M45-LpRalF (Figure 7A). Likewise, inefficient Arf1-GFP recruitment to the LCV was observed for both M45-RpRalF_1–342SS_ and the M45-LpSec7-RpCD chimeric protein having the Sec7 region from LpRalF fused to the translocation-competent capping domain from RpRalF_1–342SS_. Thus, these data show that the Sec7 domain of RpRalF is as efficient as that of LpRalF in activating Arf1 *in vivo*. They also indicate that the capping domain regulates the efficiency of Arf1 recruitment to the LCV when the protein is delivered into host cells by the Dot/Icm system.

The LCV originates as a plasma membrane-derived organelle that matures through a process involving fusion with ER-derived vesicles [17], [19]. Given that the RpRalF capping domain can associate with lipids at the plasma membrane and the LpRalF capping domain interacts with determinants on the ER and early secretory vesicles, we asked whether the kinetics of RalF-mediated Arf1 recruitment to the LCV correlated with the kinetics by which this organelle changes identity from a plasma membrane-derived compartment to an ER-derived vacuole (Figure 7B). Arf1-GFP recruitment to the LCV increased over the first 2 hours of infection with *Legionella* producing LpRalF, and Arf1-GFP staining of the LCV remained high until at least 4 hours post-infection. For *Legionella* producing RpRalF_1–342SS_ the highest percentage of vacuoles having detectable levels of Arf1-GFP were observed at 30 min post-infection, and Arf1-GFP levels then began to drop to the point where almost no Arf1-GFP staining was observed on vacuoles after 4 hours of infection. These data suggest that RpRalF functions more efficiently on the plasma membrane-derived LCV, whereas, LpRalF functions more efficiently on the late ER-derived LCV, which correlates with the localization properties of the respective capping domains.

## Discussion

This study demonstrates that both *Legionella pneumophila* and *Rickettsia prowazekii* encode related RalF proteins with a functional Sec7 domain that stimulates the exchange of GDP for GTP on the host protein Arf1. *In vitro* studies indicate the Sec7 domain in both RalF proteins display similar catalytic activities. Arfs control distinct processes on different organelles in eukaryotic cells through their recruitment to different membranes in a process that is coincident with their activation. ArfGEFs have structural determinants lying outside of the Sec7 domain that control Arf-dependent activities and influence the locations of both the ArfGEF and consequently the recruited Arf [7]–[10]. This is likely the reason for the presence of at least fifteen different human proteins containing Sec7 domains to control the activities of only five Arf GTPases [5]. Data presented here indicate that the bacterial RalF proteins have also diverged to control Arf-dependent processes on distinct organelles, and that changes in the determinants that mediate GEF localization are involved in conferring differences in RalF protein function. Interestingly, these localization determinants appear to lie outside of the Sec7 domain itself. Thus, there are important parallels between the mechanisms that drive functional divergence of eukaryotic Sec7 proteins and bacterial Sec7 proteins, and understanding the function of these bacterial Arf GEFs could provide new insight into the key determinants that enable Sec7 domain proteins to control distinct Arf-dependent cellular processes.

The capping domain in RalF is the primary determinant that mediates spatial regulation of RalF activity. The capping domain has the ability to interact with cellular membranes and to inhibit GEF activity by interacting with the Sec7 domain. Although the LpRalF and RpRalF capping domains display high homology, these regions have different membrane-binding properties. The LpRalF capping domain localizes to membranes of the host secretory pathway by a mechanism that appears to be independent of a specific charged lipid signature and may involve protein-protein interactions. This suggests that LpRalF may play a role after the plasma membrane-derived vacuole containing *Legionella* has initiated interactions with host vesicles derived from the ER. Consistent with this hypothesis, it has been shown that Arf is not essential for the interaction of early secretory vesicles that exit the ER with the vacuole containing *Legionella*, but is important for fusion of membranes containing resident ER proteins with the vacuole containing *Legionella* [19]. Thus, LpRalF could facilitate this second stage of vacuole maturation through the activation of Arf on the LCV after initial fusion events have occurred with the early secretory vesicles.

Data indicating that the RpRalF protein interacts with the host plasma membrane and has the capacity to modulate actin dynamics would be consistent with the intracellular lifestyle of *Rickettsia prowazekii*, which is a pathogen that rapidly lyses the endocytic vacuole after uptake by host cells and replicates in the cytosol [24], [25]. Although it has not been conclusively demonstrated that RpRalF is delivered into host cells during infection, *R. prowazekii* encodes a type IVA secretion apparatus [30] and the RpRalF protein has been proposed to be a substrate for this system [24]. One possibility is that RpRalF is delivered upon contact with host cells, where it may then participate in the endocytosis process that enables the pathogen to invade host cells. Another possibility is that RpRalF is delivered into host cells after the bacteria has lysed its vacuole, and by activating Arf at the plasma membrane RpRalF helps to maintain the architecture of the cell by stabilizing either the actin network or the integrity of the plasma membrane. We found that overexpressed YFP-RpRalF localizes to actin stress fibers in HeLa cells. Stress fibers are connected to the plasma membrane at focal adhesions [31], and Arf signaling events at focal adhesions are known to play a role in modulating stress fiber dynamics [32], [33]. Thus, this localization of YFP-RpRalF to both the plasma membrane and stress fibers could reflect the ability of RpRalF to link Arf signaling at the plasma membrane to actin-dependent cytoskeletal dynamics. This localization is consistent with a functional link between RpRalF and stress fiber formation, however, it should be noted that the colocalization between overproduced YFP-RpRalF and stress fibers may not be representative of the normal distribution of RpRalF in cells during infection, which remains to be determined. Although studies to determine the role of RpRalF during infection are complicated by the inability to culture *Rickettsia* outside of eukaryotic host cells and classification of this organism as a select agent, these data indicate that cell biological and biochemical analysis can be used to better understand how this protein functions.

Overexpression of the capping domain from LpRalF and RpRalF was found to interfere with distinct Arf-mediated processes in the cell. This “dominant-negative” effect suggests that the overexpression of the capping domain may titrate a limiting factor needed for homeostatic regulation of the respective pathways targeted by these bacterial proteins. Importantly, the wild-type LpRalF protein containing a functional GEF domain displayed a dominant-negative phenotype on the host secretory pathway that was similar to the effect of the catalytically-inactive LpRalF_E103A_ protein or the LpRalF capping domain alone. This implies that LpRalF is not a functional mimic of a host GEF that regulates membrane transport in the secretory pathway, and suggests that the capping domain subverts host determinants important for regulation of membrane transport in the early secretory pathway to control GEF activity during infection. The dominant-negative phenotype observed upon overexpression of LpRalF is not observed during infection, which is likely because the amount of LpRalF translocated during infection is well below the number of molecules that would be needed to titrate an essential host factor.

Expression of wild type RpRalF appeared to maintain stress fibers, whereas, the catalytically inactive RpRalF_E100A_ protein or RpRalF_189–458_ functioned as dominant-negative proteins. This suggests that RpRalF subverts Arf function at the plasma membrane to enhance cell stability by acting in a manner that would be similar to an endogenous GEF, such as members of the cytohesin family of Arf GEFs that include the protein ARNO (cytohesin 2). Recent studies suggest that ARNO plays a role in promoting cell migration by coupling Arf activation to events that include recycling of cell adhesion molecules such as integrins to the plasma membrane and activation of factors such as CDC42 that promote actin-dependent cell movement, whereas, GRP1 (cytohesin 3) appears to have the opposite effect [34], [35].

Similar to what is shown here for RalF, the GEF activity of cytohesin proteins is autoinhibited. For the cytohesins, autoinhibition is mediated by a polybasic region and a linker that act as pseudo-substrates that bind the Sec7 domain [36]. Autoinhibition of the GEF activity is relieved by a feed forward loop that involves interactions between the cytohesin pleckstrin homology domain and linker domain with phospholipids and active Arf at the plasma membrane [36], [37]. Thus, it is possible that the capping domain, in association with the tail domain in RpRalF, binds to components that are required for regulating cell adhesion by the cytohesin proteins, explaining why overexpression of this C-terminal RpRalF region disrupts regulation of cytohesin-mediated processes in the cell. If RpRalF retains GEF activity, however, overexpression does not have a dramatic effect on cell morphology, maybe because in this case the effector is able to duplicate the activity of the endogenous GEF that is not able to function due to the titrating activity of the RpRalF C-terminal region.

In summary, these data indicate that the LpRalF and RpRalF proteins are both effectors capable of modulating host cellular processes by functioning as GEFs for Arf GTPases. RalF regulation is mediated by a C-terminal capping domain that controls both localization and enzymatic functions of the protein. These data suggest a model whereby interactions between the RalF C-terminal domains and host membrane-bound determinants lead to a conformational change in the protein that disengages the capping domain from the Sec7 interface, and this enables the RalF proteins to activate Arf by promoting nucleotide exchange. A major objective is to characterize RalF C-terminal domain interaction with host determinants. This information should lead to a much better understanding of how these bacterial effectors are regulated *in vivo* and how Arf manipulation by these effectors may enhance intracellular survival and replication of two pathogens having dramatically different intracellular lifestyles.

## Materials and Methods

### Bacteria and DNA constructs

*Escherichia coli* DH5α and BL21 strains were cultivated in Luria-Bertani (LB) media with antibiotics when necessary at the following concentrations: ampicillin, 100 µg/ml ; kanamycin, 30 µg/ml. *Legionella pneumophila* serogroup 1, strain Lp01 [38], and the *ΔralF* mutant [21] were used for infection experiments. *Legionella* strains were grown on charcoal yeast extract (CYE) plates (1% yeast extract, 1% *N*-(2-acetamido)-2-aminoethanesulfonic acid (ACES; pH 6.9), 3.3 mM l-cysteine, 0.33 mM Fe(NO_3_)_3_, 1.5% bacto-agar, 0.2% activated charcoal), supplemented with 10 µg/mL chloramphenicol when required [39].

Different constructs of *L. pneumophila* and *R. prowazekii ralF* genes were amplified from genomic DNA as EcoRI/BamHI (LpRalF_1–374_, LpRalF_192–374_, RpRalF_189–458_, RpRalF_189–359_ and RpRalF_359–459_), PstI/BamHI (LpRalF_1–201_) or BglII/EcoRI (RpRalF_1–458_) fragments and were cloned into pEYFP-C1 vector. To obtain MBP-tagged proteins, ARNO, LpRalF, RpRalF, LpRalF_1–201_, RpRalF_1–203_, LpRalF_192–374_ and RpRalF_189–359_ encoding genes were cloned in pMalc5x vector at EcoRI/BamHI sites. For the RpRalF full length protein, a codon-optimized gene for expression in *E. coli* synthesized by Genscript was used. For expression by *L. pneumophila*, pJB1806 vector [40] was used that had an IcmS promoter and an M45 tag sequence cloned at the EcoRI/BamHI sites upstream of inserted genes. Genes encoding LpRalF_1–374_, RpRalF_1–342SS_ and the chimeric proteins RpSec7-LpCD and LpSec7-RpCD were cloned in pJB1806 at BamHI/SalI sites. The codon-optimized *R. prowazekii ralF* gene was used. The chimeric genes were obtained by overlapping PCR generating RpRalF_1–342_-LpRalF_340–374_ (RpRalF_1–342SS_), RpRalF_1–195_-LpRalF_198–374_ (RpSec7-LpCD) and LpRalF_1–197_-RpRalF_196–342_-LpRalF_340–374_ (LpSec7-RpCD) flanked by BamHI/SalI sites, subsequently inserted in pJB1806. Site-directed mutagenesis was performed to obtain single point mutants. Plasmids were amplified using two complementary primers containing the desired mutation with Pfu turbo (Stratagene). The result was digested by DpnI for 1 h at 37°C before transformation in DH5α.

### Antibodies and reagents

The mouse monoclonal PDI antibody was purchased from Stressgen Biotechnologies Corporation. The GM130 antibody is a mouse monoclonal antibody from BD Biosciences Pharmingen. The polyclonal GFP rabbit antibody is from Invitrogen. The Texas Red X phalloidin was purchased from Molecular Probes. The plasmid encoding RFP-PALM is from Clontech.

The complete EDTA-free protease inhibitor cocktail and the FuGene6 reagent were purchased from Roche Applied Science. Lipofectamine 2000 was purchased from Invitrogen. The Phospha-light SEAP kit was purchased from Applied Biosystem.

### Protein purification

*E. coli* DH5α cells transformed with plasmids encoding MBP-RalF, MBP-RalF_Sec7_, MBP-RalF_capping domain_ or MBP-ARNO proteins were grown at 37°C in LB broth containing appropriate antibiotic to an optical density >0.5. IPTG was added to the medium at a final concentration of 1 mM and samples were incubated for 3 h at 37°C with shaking. Cells were harvested by centrifugation 10 min at 6000 rpm, resuspended in lysis buffer (100 mM NaCl, 1 mM MgCl2, 20 mM HEPES pH 7.4, 2 mM EDTA, 1 mM PMSF, 1 mM DTT, 1% triton) and sonicated 3 times for 1 min. Soluble fraction was obtained by centrifugation 20 min at 15000 rpm. The lysate was mixed with 1 mL of amylose resin (New England Biolabs), and incubated 1 h30 at 4°C with shaking. The resin was then washed three times with wash buffer (100 mM NaCl, 1 mM MgCl2, 20 mM HEPES pH 7.4), and eluted in elution buffer (100 mM NaCl, 1 mM MgCl2, 20 mM HEPES, pH 7.4 and 50 mM maltose) 30 min at 4°C. Eluted protein was collected by centrifugation, dialyzed and stored at −70°C before use.

*E. coli* BL21 cells transformed with plasmids encoding 6xHis-ΔN17Arf1 or 6xHis-ΔN12Arf6 proteins were grown at 37°C in LB broth containing appropriate antibiotic to an optical density >0.5. IPTG was added to the medium at a final concentration of 0.2 mM and samples were incubated for 6 h at 30°C with shaking. Cells were harvested by centrifugation 10 min at 6000 rpm, resuspended in lysis buffer +10 mM imidazole and sonicated 3 times for 1 min. Soluble fraction was obtained by centrifugation 20 min at 15000 rpm. The lysate was mixed with 1 mL of Ni-NTA agarose (Qiagen), and incubated 1 h 30 min at 4°C with shaking. The resin was then washed three times with wash buffer +20 mM imidazole, and eluted in buffer (100 mM NaCl, 1 mM MgCl2, 20 mM HEPES, pH 7.4 and 200 mM imidazole) 30 min at 4°C. Eluted protein was collected by centrifugation, dialyzed and stored at −70°C before use.

### Nucleotide exchange assay

Purified 6XHis-tagged ΔN17Arf1 or ΔN12Arf6 proteins were incubated in loading buffer (20 mM Tris, pH 8.0, 100 mM NaCl, 5 mM EDTA, and 1 mM DTT) containing a 10 fold molar excess of *N*-methylanthraniloyl (mant)-GDP (Invitrogen) for 30 min at 37°C. To terminate the loading reaction, MgCl_2_ was added to a final concentration of 10 mM, and free mant-GDP was removed using a polyacrylamide Desalting Column (Piercenet), previously equilibrated with column buffer (20 mM Tris, pH 8.0, 100 mM NaCl, 2 mM MgCl2).

Exchange kinetics were monitored using the decrease in emission intensity accompanying release of mant-GDP as described in DiNitto *et al* [36]. Exchange reactions were initiated by mixing mant-GDP loaded ΔN17Arf1 or ΔN12Arf6 at a final concentration of 2 µM with varying concentrations of the GEF in the presence of 200 µM GTP. Data were collected with an Infinite M1000 microplate reader (Tecan) using excitation and emission wavelengths of 360 nm and 440 nm. K_cat_/K_m_ values were calculated as in DiNitto *et al* [36].

### Cell culture and transient transfections

HEK293 and Hela cells were maintained in minimal Dulbecco's Modified Eagle's Medium, supplemented with 10% heat inactivated fetal bovine serum, 100 µg/mL penicillin and 10 µg/mL streptomycin at 37°C with 5% CO_2_. Chinese Hamster Ovary (CHO) cells were maintained in minimal αMEM, supplemented with 10% heat inactivated fetal bovine serum, 100 µg/mL penicillin and 10 µg/mL streptomycin at 37°C with 5% CO_2_.

For transfection, Hela and CHO cells were plated at a density of 6.10^4^ cells per well in 24-well tissue culture plates with glass coverslips and transfected the following day using Fugene6. HEK293 cells were plated at a density of 1.10^6^ cells per well in 6-well tissue culture plates and transfected the following day using lipofectamine2000.

### PDI and GM130 Immunofluorescent staining and cell imaging

After transfection, cells were incubated for 24 hours then fixed with 3% PFA for 20 min at room temperature (RT). Cells were permeabilized in Blocking Buffer (0.2% saponin, 0.5% BSA, 1% fetal calf serum in PBS) for 20 min. Coverslips were then washed with PBS and incubated with primary antibody (mouse anti-GM130 1/1000 or mouse anti-PDI 1/500) diluted in Blocking Buffer for 1 h at RT. Cells were then washed with PBS and incubated with an Alexa Fluor 594 anti-mouse antibody (Invitrogen) at a dilution of 1/250 in Blocking Buffer for 1 h at RT. Finally, cells were washed in PBS and mounted on plain microscope slides. Cells were subsequently visualized by fluorescence microscopy using a Nikon Eclipse TE2000-S microscope and a 100×/1.40 oil objective (Nikon Plan Apo). Z-stacks were acquired using a Hamamatsu ORCA-ER camera and 3D max was generated. Images were exported to Image J and deconvoluted for the production of figures. Confocal images were obtained using a Zeiss LSM 510 Laser Scanning Microscope.

### Cell fractionation

HEK293 cells were grown in 6-well plate and transfected with YFP or YFP-RalF encoding plasmids. 24 h after transfection, cells were washed once with PBS and collected in PBS. Cells were then centrifuged at 1,000 rpm for 2 min. Cell pellets were resuspended in 200 µL homogenization buffer (150 mM KCl, 20 mM HEPES pH 7.4, 2 mM EDTA) and passed 12 times in a 27G-needle. The lysate was centrifuged at 3000 rpm for 5 min at 4°C to remove nuclear fraction. The supernatant was collected and centrifuged at 100,000 g for 1 h at 4°C. The supernatant (soluble fraction) was collected and the pellet (insoluble fraction) was resuspended in 200 µL homogenization buffer. Both fractions were separated by SDS-PAGE and blotted with anti-GFP antibody. Anti-calnexin and anti-α-tubulin antibodies were used as markers of the membrane and the cytosol fraction, respectively.

### Membrane strip assay

RalF capping domain affinity to lipids was assessed using commercially available membrane lipid strips (Echelon). Membranes spotted with 100 pmol of fifteen different biologically important lipids found in cell membranes were blocked with 2% fat free milk in TBST (50 mM Tris, 150 mM NaCl, 0.1% Tween-20 (v/v), pH 7.4) for 1 h at room temperature prior to incubation with the MBP fusion proteins (1 µg/mL) 1 h at room temperature. Binding of the fusion proteins to lipids was visualized by chemiluminescence (ECL, Perkin Elmer), using a polyclonal anti-MBP antibody (New England Biolabs) and a goat anti-rabbit peroxidase-labeled antibody (Invitrogen).

### Secretion assay

CHO cells were plated in 24-well dishes at 3.10^4^ cells per well. After 18 hours incubation, the cells were co-transfected with 200 ng of plasmid encoding the indicated YFP-tagged protein and 300 ng of a plasmid encoding a secreted alkaline phosphatase (SEAP) protein. 24 hours after transfection, cells were washed, and fresh tissue culture medium was added. SEAP activity was measured 7 hours later, in the supernatant and in cells, using the Phosphalight SEAP kit (Applied Biosystems). The ratio of SEAP activity detected in the culture medium to the cells-associated SEAP activity is measured. Data are then normalized and compared to control cells, expressed as percent of control cell activity.

### Actin filaments examination

24 hours after transfection, cells were fixed with 3% PFA for 20 min at RT. Cells were permeabilized with 0.1% triton in PBS for 5 min, and blocked in Blocking Buffer (0.5% BSA, 1% fetal calf serum in PBS) for 30 min. Coverslips were then incubated for 1 h at RT in Blocking Buffer supplemented with TexasRed conjugated phalloidin at a concentration of 82.5 nM. Finally cells were washed with PBS and mounted on plain microscope slides before examination by immunofluorescence microscopy.

### *Legionella* infections

*Legionella* were harvested from 2-day heavy patch, and used to infect HEK293 cells stably expressing Arf1-GFP and the receptor FcγRII. This receptor allows *L. pneumophila* opsonized with anti-*Legionella* antibodies to be internalized efficiently by non-phagocytic cells [41]. Bacteria were opsonized with rabbit anti-*Legionella* antibody diluted 1/1000 for 30 min at 37°C. Bacteria were then added to the cells at an MOI of 1. The cells were centrifuged 5 min at 1000 rpm and incubated for the desired time at 37°C. Cells were then fixed with PFA for 20 min at room temperature, and stained for extracellular bacteria with blue anti-rabbit antibodies. Permeabilization was performed by a methanol treatment 1 min at RT before staining total bacteria with red anti-rabbit antibodies. The number of LCVs positive for Arf1-GFP was quantified.

### Statistics

Differences between control and experimental groups were analyzed by Student's T-test. Threshold P-value below the statistical significance value of 0.01 are presented in figures.

## Supporting Information

Figure S1 Expression and translocation of proteins used in the complementation studies. **A**) Western Blot α-M45 on *Legionella* Δ*ralF* crude extracts expressing M45-tagged LpRalF, RpRalF_1–342SS_, LpSec7-RpCD or RpSec7-LpCD. **B**) HEK293-FcγRII cells were infected with indicated *L. pneumophila* strains carrying a plasmid encoding the indicated Cya fusion proteins. cAMP level in the cell cytosol was quantified 1 h post-infection. Average and standard deviation were obtained from three independent samples.
(TIF)

Protocol S1 Describes methods related to Figure S1.
(DOCX)

Video S1 Ectopic expression of YFP-RpRalF_189–458_ leads to cell lysis. Hela cells stably expressing the actin marker RFP-lifeactin (kind gift from Dr. Herve Agaisse) were transfected with a plasmid encoding YFP-RpRalF_189–458_. 7 h after transfection, cells starting expressing YFP were imaged using a spinning disk confocal microscope overnight. Data were analyzed with Volocity software.
(AVI)
