# Supplementary figures and images for: The Capping Domain in RalF Regulates Effector Functions
Source: PLoS Pathog. 2012 Nov 15;8(11):e1003012. doi: 10.1371/journal.ppat.1003012 (PMC3499574; doi:10.1371/journal.ppat.1003012)

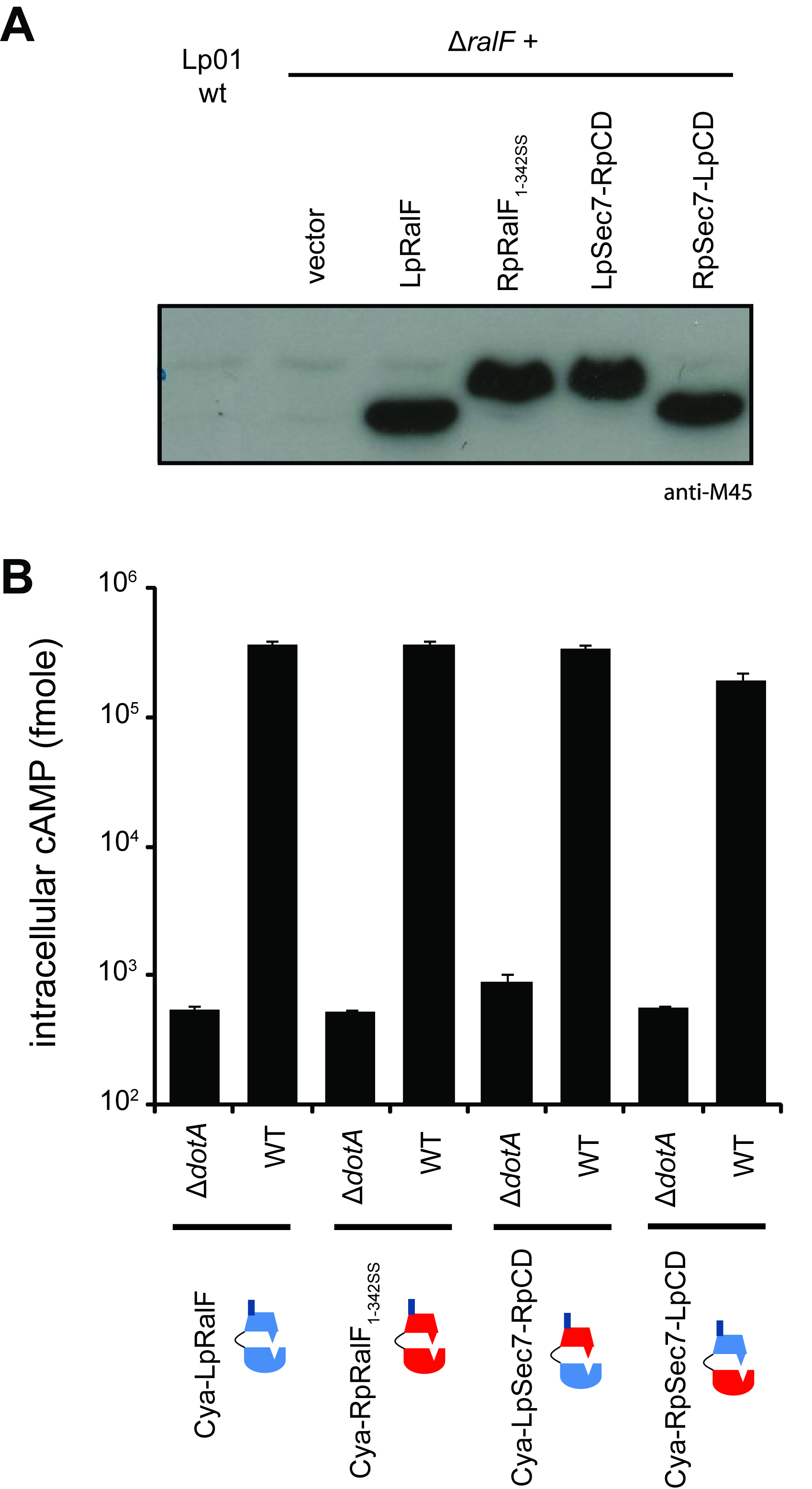

Supplement: Figure S1 — Expression and translocation of proteins used in the complementation studies. A) Western Blot α-M45 on Legionella ΔralF crude extracts expressing M45-tagged LpRalF, RpRalF1–342SS, LpSec7-RpCD or RpSec7-LpCD. B) HEK293-FcγRII cells were infected with indicated L. pneumophila strains carrying a plasmid encoding the indicated Cya fusion proteins. cAMP level in the cell cytosol was quantified 1 h post-infection. Average and standard deviation were obtained from three independent samples. (TIF) [file ppat.1003012.s001.tif]
