# Supplementary material for: The Capping Domain in RalF Regulates Effector Functions
Source: PLoS Pathog. 2012 Nov 15;8(11):e1003012. doi: 10.1371/journal.ppat.1003012 (PMC3499574; doi:10.1371/journal.ppat.1003012)
Supplement: Protocol S1 — Describes methods related to Figure S1. (DOCX) [file ppat.1003012.s002.docx]

Generation of Cya-RalF constructs

CYA_2-405_ domain was amplified generating an EcoRI/BamHI fragment. This fragment was introduced at EcoRI/BamHI sites in pJB1806-RalF plasmids, thereby removing the IcmS promoter.

Cya translocation assay : evaluation of LpRalF, RpRalF_1-342SS_, LpSec7-RpCD and RpSec7-LpCD translocation during infection

Translocation of Cya-fused proteins into HEK293-FcγRII cells after infection with *Legionella* was assayed as described previously [[1](#_ENREF_1)] with minor modifications. Briefly, HEK293-FcγRII cells were seeded in 24-well plates, and infected by *Legionella* strains expressing Cya fusions at a multiplicity of infection of 30. 1h post-infection, infected cells were lysed in 200 μl lysis buffer (50 mM HCl, 0.1% triton-X100); cAMP levels were determined using the cAMP Biotrak Enzymeimmunoassay System (GE Healthcare, RPN225) according to manufacturer’s instructions.

1. Nagai H, Cambronne ED, Kagan JC, Amor JC, Kahn RA, et al. (2005) A C-terminal translocation signal required for Dot/Icm-dependent delivery of the *Legionella* RalF protein to host cells. Proc Natl Acad Sci U S A 102: 826-831.
